# Complete Genome Sequence of Rhodococcus erythropolis Phage Shuman

**DOI:** 10.1128/MRA.00113-19

**Published:** 2019-03-28

**Authors:** Sucely Ponce Reyes, Peter J. Park, Daniel Kaluka, Jacqueline M. Washington

**Affiliations:** a Department of Natural Sciences, Nyack College, Nyack, New York, USA; Georgia Institute of Technology

## Abstract

Shuman is a bacteriophage isolated in Nyack, New York, using Rhodococcus erythropolis NRRL B-1574 as a host. It is a member of cluster CA and has a genome length of 46,544 bp. Shuman contains 67 predicted protein-coding genes, 3 tRNA genes, and no transfer-messenger RNA (tmRNA) genes.

## Abstract (precis)

Shuman is a bacteriophage isolated in Nyack, New York, using Rhodococcus erythropolis NRRL B-1574 as a host. It is a member of cluster CA and has a genome length of 46,544 bp.

## ANNOUNCEMENT

Shuman is a bacteriophage that was isolated from soil collected at the Nyack College campus in Nyack, New York, in 2017 by using Rhodococcus erythropolis NRRL B-1574 as a host. The phage was isolated by direct plating at 30°C on peptone-yeast extract-calcium (PYCa) medium supplemented with 0.1% dextrose and forms turbid plaques 1 to 2 mm in diameter. Briefly, soil was incubated with PYCa to obtain a soil extract which was filtered through a 0.22-µm filter, and 50 µl of this extract was added to 250 µl late-exponential-phase Rhodococcus erythropolis and plated with 3 ml molten PYCa top agar (0.35%). To purify, well-isolated individual plaques were picked and added to phage buffer with glycerol (10 mM Tris-HCl [pH 7.5], 10 mM MgSO_4_, 1 mM CaCl_2_, 68 mM NaCl, and 10% glycerol), serially diluted, and replated for three successive rounds.

DNA was isolated from a high-titer lysate of purified phage by using a Promega Wizard DNA extraction kit. A sequencing library was prepared from genomic DNA by using an NEB Ultra II FS kit with dual-indexed barcoding. It was multiplexed with 47 other phage genome libraries and run on an Illumina MiSeq instrument, yielding ∼555,000 single-end 150-base reads from the Shuman sample, representing ∼1,684-fold coverage of the genome. These raw reads were assembled using Newbler version 2.9 with default settings, yielding a single-phage contig (46,544 bp) which was checked for completeness, accuracy, and phage genomic termini using Consed version 29 and as previously described (1). The Shuman genome is 46,544 bp in length, with 58.6% GC content, and has defined ends with 10-base 3′ single-stranded extensions (CGGCCGTGAT). Annotation analysis was performed using the following databases and software as of July 2018 with default settings: DNA Master (http://cobamide2.bio.pitt.edu/computer.htm), PECAAN (https://pecaan.kbrinsgd.org/), Phamerator (2), Glimmer 3.0 (3), Genemark 2.5 (4), HHPRED (5), NCBI BLAST 2.7 and Conserved Domain Database at NCBI (6), ARAGORN version 1.2.38 (7), tRNA scan-SE 3.0 (8), and TMHMM 2.0 (9, 10).

Based on >90% nucleotide similarity across the entire genome, Shuman is classified as a member of cluster CA, the most common cluster containing *Rhodococcus* phages, to which 70% of the sequenced *Rhodococcus* phages belong (11). Nearly all cluster CA phages have been isolated using Rhodococcus erythropolis RIA 643. Cluster CA phages are members of the *Siphoviridae* family and are predicted to be temperate.

Sixty-seven protein-coding genes and 3 tRNAs were identified in the Shuman genome. Similarly to the other cluster CA phages, ∼50% of the predicted protein-coding genes were assigned functions, and the tRNAs are within 2 kb of the left end of the genome (12). Four genes with no known function are predicted to form transmembrane helices. No transfer-messenger RNAs (tmRNAs) were identified. Shuman is most similar to StCroix (GenBank accession number MF324900), which was isolated in 2014 in Hudson, Wisconsin, with 98% identity over 99% coverage. The Shuman genome is arranged with the structural and assembly genes on the left end while the regulatory and replication genes are on the right end of the genome.

### Data availability.

Bacteriophage Shuman genome sequence is available at GenBank under the accession number MH316569. The raw sequences are available in the NCBI SRA database under the accession number SRR8477199.
